# Endoscopic resection of duodenal bulb neuroendocrine tumor larger than 10 mm in diameter

**DOI:** 10.1186/1471-230X-11-67

**Published:** 2011-06-10

**Authors:** Shozo Yokoyama, Katsunari Takifuji, Masaji Tani, Manabu Kawai, Teiji Naka, Kazuhisa Uchiyama, Hiroki Yamaue

**Affiliations:** 1 Second Department of Surgery, Wakayama Medical University, School of Medicine, Wakayama, Japan

## Abstract

**Background:**

Endoscopic treatment for duodenal bulb neuroendocrine tumor larger than 10 mm is still controversial. This report presents four cases successfully treated with endosonography (EUS)-assisted endoscopic mucosal resection (EMR) procedure for duodenal bulb neuroendocrine tumor larger than 10 mm in diameter.

**Methods:**

The case series of four patients diagnosed with neuroendocrine tumor from 2003 to 2008 were reviewed. EUS demonstrated well-defined hypoechoic tumors confined to the submucosal hyperechoic layer and the underlying hypoechoic muscularis propria was intact in all four patients. EMR were planned and performed for the duodenal bulb neuroendocrine tumors larger than 10 mm.

**Results:**

En bloc resections with tumor free lateral and basal margins were accomplished using an endoscopic diathermic snare with forward-viewing instruments without any complications. Neither residual duodenal neuroendocrine tumors nor metastatic lesions were detected during the observation period ranging 19 to 78 months

**Conclusion:**

Duodenal bulb neuroendocrine, larger than 10 mm in diameter, can be treated by endoscopic procedure, after confirming that the tumor confined to the submucosal layer in EUS examination, and no lymph node involvement by abdominal CT and US.

## Background

Recently, endoscopic resection of neuroendocrine of the duodenum is increasingly performed as an alternative to conventional surgery. Most reports state that duodenal neuroendocrine tumor can be treated by endoscopic excision, when the diameter is less than 10 mm and there is no invasion of the muscularis propria [1]. On the other hand, others have shown that the features associated with metastatic risk are a diameter greater than 2 cm [2], and those with duodenal neuroendocrine tumor with a diameter larger than 10 mm can be successfully treated by endoscopic procedure [3,4]. Therefore, it is controversial whether endoscopic treatment is appropriate for duodenal neuroendocrine tumor larger than 10 mm in diameter. This report describes the successful EUS-assisted EMR for duodenal bulb neuroendocrine tumors larger than 10 mm in diameter.

## Methods

**Figure 1:**
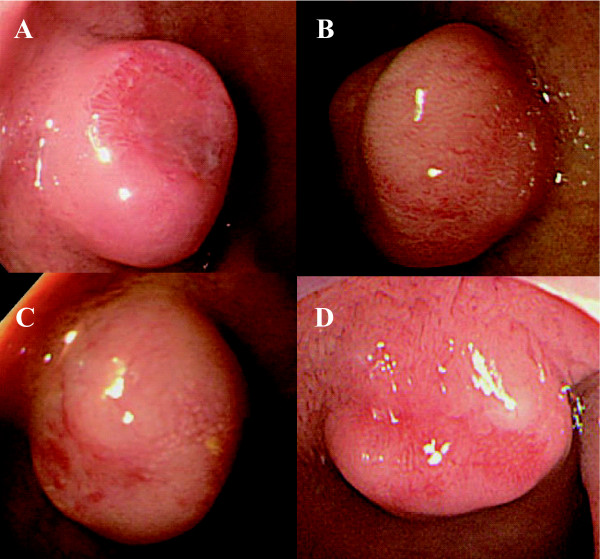
**Endoscopic findings of the duodenal neuroendocrine tumors**. **A, B, C, D**. Semipedunculated elevation accompanied by an irregularly shaped erythematous central depression (**A**: patient 1; **B**: patient 2; **C**: patient 3; **D**: patient 4).

**Figure 2:**
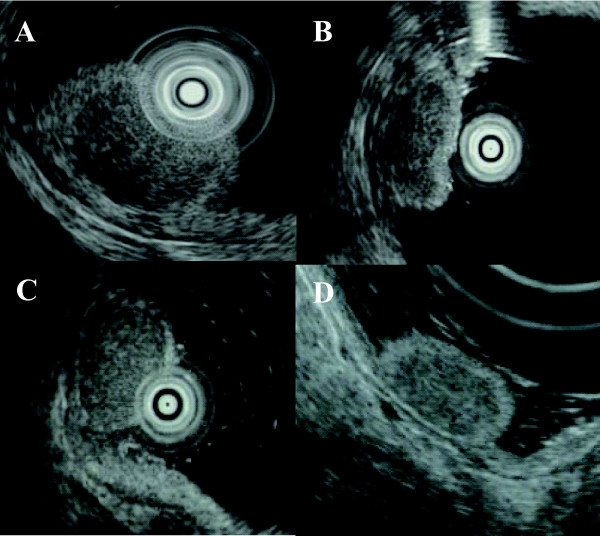
**Endosonographic findings of the duodenal neuroendocrine tumors**. **A, B, C, D**. Well-defined hypoechoic tumor confined to submucosal hyperechoic layer and the intact underlying hyperechoic muscularis propria (**A**: patient 1; **B**: patient 2; **C**: patient 3; **D**: patient 4).

The case series of four patients diagnosed with neuroendocrine tumor from 2003 to 2008 were reviewed. All four cases had no symptoms for the tumor. All of them were evaluated by endoscopic and endosonographic examination of the upper gastrointestinal tract, whole abdominal computed tomography (CT), and abdominal ultrasonography (US). Endoscopic findings showed that all four cases were characterized by semipedunculated elevation accompanied by irregularly shaped erythematous central depression (Figure 1). EUS demonstrated that well-defined hypoechoic tumors confined to the submucosal hyperechoic layer, and that the underlying hypoechoic muscularis propria was intact in all four patients (Figure 2). No other polypoid lesions were found in the gastrointestinal tract, and no enlarged lymph nodes were identified in any of the patients on abdominal CT and US. The duodenal tumors were diagnosed histologically as neuroendocrine tumors after endoscopic biopsy with the standard forceps method. The surgical indications for duodenal bulb neuroendocrine tumor larger than 10 mm in diameter were explained to all of the patients and all provided informed consent for endoscopic resection. Thereafter, EMR were planned and performed for the duodenal bulb neuroendocrine tumors.

## Results

These lesions were fully lifted with submucosal injection of saline. En bloc resections with tumor free lateral and basal margins were accomplished using an endoscopic diathermic snare with forward-viewing instruments (Olympus GIF 2T-260) without any complications (Table 1). The histopathological examination confirmed the diagnosis of neuroendocrine tumors, which consisted of small uniform cells arranged in a ribbon- like pattern with no mitosis, confined to submucosal layer for patient 1, 2 and 3, and confined to mucosal layer in patient 4 (Table 2). All four tumors were endocrinologically inactive, and diagnosed as neuroendocrine tumor with positive immunohistochemical staining for chromogranin A. These patients were followed up annually by endoscopy with biopsies and abdominal CT and US. Neither residual duodenal neuroendocrine tumors nor metastatic lesions were detected during the observation period ranging 19 to 78 months (Table 2).

**Table 1:** Endoscopic procedure and complications

Case	Endoscopic procedure	Duration of Surgery (minutes)	Perforation	Bleeding
1.	diathermic snare	15	No	No
2.	diathermic snare	20	No	No
3.	diathermic snare	17	No	No
4.	diathermic snare	15	No	No

**Table 2:** Characteristics of four duodenal cartinoid tumor

Case	Location (bulbus)	Size (mm)	Depth	lymphatic/ venous invasion	mitosis	follow-up (months)	recurrence/ metastasis
1.	anterior	12	sm	No/No	No	78	No/No
2.	posterior	13	sm	No/No	No	56	No/No
3.	anterior	11	sm	No/No	No	43	No/No
4.	superior	12	m	No/No	No	19	No/No

## Discussion

Duodenal neroendocrine tumor, formerly termed as carcinoid tumor, is rare entity. The tumors is derived from enterochromaffin cells, and often occurred in gastrointestinal tract. Only 2.6% duodenal carcinoid tumors is reported of all carcinoid tumors in the United States[5]. The rarity makes difficulty to address the feasibility of the endoscopic treatment for this tumor. Although larger study is needed to confirm the indication of endoscopic procedure for the tumor, small case series like our study is also important for uncommon tumor.

Surgical resection is generally recommended for neuroendocrine tumor due to the risk of lymph node involvement [6]. Endoscopic treatment such as EMR or endoscopic submucosal dissection is an alternative to conventional surgery including a duodenal resection in patients with neuroendocrine tumor of the duodenum, since recent development of endoscopic technology makes it possible to detect small and early neuroendocrine tumors. EMR technique was successful and safe for most small duodenal bulb neuroendocrine tumor[1,7-10]. Some report mentioned that EMR procedure is also useful for neuroendocrine tumor located on the second portion[11]. Although more case series for duodenal neuroendocrine tumor is needed, EMR technique is a safety procedure for small neuroendocrine tumors.

The appropriate indication criteria are needed to perform endoscopic treatment, because neuroendocrine tumor is considered to have malignant potential for metastasis. Burke described that the features associated with increased risk of metastasis included involvement of the muscularis propria, size larger than 20 mm, and the presence of mitotic figures [2]. Duodenal neuroendocrine tumors smaller than 20 mm can be treated safely with local excision alone [12]. The metastatic rates of neuroendocrine tumor are proportional to the size of the tumor: 8.3% with tumors smaller than 5 mm, 10.5% with tumors between 5.1 and 10 mm, 17.4% with tumors between 10.1 and 15 mm, and 8.8% with those between 15.1 and 20 mm [13]. Therefore, if neuroendocrine tumor smaller than 20 mm in diameter is diagnosed to be localized within the submucosal layer by EUS examination, endoscopic resection may be one of the option for the treatment.

Preoperative EUS, examining not only the depth of tumor but also lymph node involvement, is an important modality for determination of endoscopic respectability, when neuroendocrine tumor larger than 10 mm in diameter is evaluated for the surgical options [7,9], since neuroendocrine tumor with invasion to the muscularis propria is associated with metastasis. Meanwhile, it is recommended that full-thickness resection of lesions in the gastrointestinal tract should be performed if the neuroendocrine tumor has extensively infiltrated the submucosal layer or involves the muscle layer, because conventional EMR does not eliminate the possibility of tumor seeding in the vertical margin due to the burning effect[14,15]. Therefore, endoscopic resection must be performed, after confirming that the tumor is confined to the submucosal layer and there is no metastasis. Moreover, endoscopic treatment must be followed by histopathological examination confirming that additional surgical treatment is required or not. In the current series, histopathological investigations supported that EUS assisted EMR can be performed safely and completely.

## Conclusions

The present report described that four patients with duodenal neuroendocrine larger than 10 mm in diameter could be endoscopically resected. There was no evidence of residual tumor and metastasis during the follow up period ranging 19 to 78 months. Further follow-up is required to address this technique is safely for long-term outcome of neuroendocrine tumors[16]. After confirming that the tumor confined to the submucosal layer by EUS examination, and no lymph node involvement by abdominal CT and US, duodenal bulb neuroendocrine tumor, larger than 10 mm in diameter, can be treated by endoscopic procedure.

## Competing interests

The authors declare that they have no competing interests.

## Authors' contributions

SY, KT and HY were the lead investigators, performed the endoscopy, clinically managed the patient, designed and interpreted the manuscript, reviewed the manuscript, and gave the final approval of the version to be published. MT, MK, TN and KU were involved in drafting the manuscript, and critically revising it. All authors read and approved the final manuscript.

## Pre-publication history

The pre-publication history for this paper can be accessed here:

http://www.biomedcentral.com/1471-230X/11/67/prepub
